# Prevalence of and Risk Factors for Trachoma in Southern Nations, Nationalities, and Peoples’ Region, Ethiopia: Results of 40 Population-Based Prevalence Surveys Carried Out with the Global Trachoma Mapping Project

**DOI:** 10.1080/09286586.2016.1247876

**Published:** 2016-12-05

**Authors:** Tesfaye Haileselassie Adera, Colin Macleod, Misganu Endriyas, Michael Dejene, Rebecca Willis, Brian K. Chu, Yohannes Letamo, Tebeje Misganaw, Tamiru Mesele, Emebet Mekonnen, Alemayehu Sisay, Yeneneh Mulugeta, Wondu Alemayehu, Khumbo Kalua, Tezera Kifle Destu, Yilikal Adamu, Jennifer L. Smith, Abu Beyene, Addisu Tadesse, Anthony W. Solomon

**Affiliations:** ^a^ ORBIS International, Addis Ababa, Ethiopia; ^b^ Clinical Research Department, London School of Hygiene & Tropical Medicine, London, UK; ^c^ Sightsavers, Haywards Heath, UK; ^d^ SNNPR Regional Health Bureau, Awassa, Ethiopia; ^e^ Michael Dejene Public Health Consultancy Services, Addis Ababa, Ethiopia; ^f^ Task Force for Global Health, Decatur, GA, USA; ^g^ The Fred Hollows Foundation Ethiopia, Addis Ababa, Ethiopia; ^h^ Berhan Public Health and Eye Care Consultancy, Addis Adaba, Ethiopia; ^i^ Blantyre Institute for Community Ophthalmology, Blantyre, Malawi; ^j^ Department of Ophthalmology, Faculty of Medicine, Addis Ababa University, Addis Ababa, Ethiopia; ^k^ Consultant Ophthalmologist

**Keywords:** Ethiopia, Global Trachoma Mapping Project, prevalence, SNNPR, trachoma, trichiasis

## Abstract

***Purpose***: We sought to estimate the prevalence of trachoma at sufficiently fine resolution to allow elimination interventions to begin, where required, in the Southern Nations, Nationalities, and Peoples’ Region (SNNPR) of Ethiopia.

***Methods***: We carried out cross-sectional population-based surveys in 14 rural zones. A 2-stage cluster randomized sampling technique was used. A total of 40 evaluation units (EUs) covering 110 districts (“woredas”) were surveyed from February 2013 to May 2014 as part of the Global Trachoma Mapping Project (GTMP), using the standardized GTMP training package and methodology.

***Results***: A total of 30,187 households were visited in 1047 kebeles (clusters). A total of 131,926 people were enumerated, with 121,397 (92.0%) consenting to examination. Of these, 65,903 (54.3%) were female. In 38 EUs (108 woredas), TF prevalence was above the 10% threshold at which the World Health Organization recommends mass drug administration with azithromycin annually for at least 3 years. The region-level age- and sex-adjusted trichiasis prevalence was 1.5%, with the highest prevalence of 6.1% found in Cheha woreda in Gurage zone. The region-level age-adjusted TF prevalence was 25.9%. The highest TF prevalence found was 48.5% in Amaro and Burji woredas. In children aged 1–9 years, TF was associated with being a younger child, living at an altitude <2500m, living in an area where the annual mean temperature was >15°C, and the use of open defecation by household members.

***Conclusion***: Active trachoma and trichiasis are significant public health problems in SNNPR, requiring full implementation of the SAFE strategy (surgery, antibiotics, facial cleanliness, and environmental improvement).

## Introduction

Trachoma is the most common infectious cause of blindness worldwide. Trachoma is thought to be endemic in at least 50 countries,^1^ and has been targeted for global elimination as a public health problem by the year 2020. An estimated 200 million people live in endemic areas and are at risk of developing trachoma-related blindness.^2^ Transmission of the causative agent, ocular strains of *Chlamydia trachomatis*, occurs from eye to eye via hands, clothing and bedding, or via mechanical transmission by eye-seeking flies.^3–5^

According to the World Health Organization (WHO) simplified trachoma grading system, trachomatous inflammation – follicular (TF) and trachomatous inflammation – intense (TI) are signs of active disease most commonly found in young children. Following repeat infections, scar tissue may form on the conjunctival surfaces of the eyelids; over time these scars can lead to in-turning of the eyelashes to the point that they abrade the surface of the globe. This is known as trachomatous trichiasis.^5,6^

Trachoma is thought to be endemic throughout Ethiopia, with the highest prevalences of both active and potentially blinding trachoma found anywhere in the world.^7^ In addition, trachoma is the country’s second most common cause of blindness overall after cataract.^8^ The Southern Nations, Nationalities, and Peoples’ Region (SNNPR) is a central-southern region of Ethiopia, which covers almost 110,931 km^2^ or 10% of the country’s land area. In 2015 it had an estimated 17.8 million inhabitants, or about a fifth of the country’s population, with more than 90% living in rural areas.^9^ The region is divided into 14 administrative zones, four special woredas (districts), and one city administration, with Hawassa as the regional capital.

Prior to 2013, there were limited woreda-level data regarding the distribution of trachoma in SNNPR. Trachoma intervention programs were already active in Wolaita and Gamo Gofa zones, and where eye care non-governmental organizations (NGOs) were working, trichiasis was commonly managed. However, large areas of the region had little health care coverage to provide intelligence on disease occurrence. The 2005–2006 National Survey on Blindness, Low Vision and Trachoma examined 5415 individuals in 33 kebeles (the lowest census administrative unit) throughout SNNPR and found an overall regional prevalence of active trachoma of 33.2% among children aged 1–9 years. Although this backed previous views that trachoma was a significant public health problem in the region, data at higher resolution were needed to make intervention decisions.

To guide control programs, WHO recommends district-level prevalence data are used, with the TF prevalence in children aged 1–9 years used to determine the need for the A, F and E components of the SAFE strategy (surgery for trachomatous trichiasis cases, antibiotic distribution to clear infection, facial cleanliness, and environmental improvement). In areas with a TF prevalence ≥10%, mass administration of azithromycin is recommended for at least 3 years, together with measures to encourage facial cleanliness and improve the sanitation provisions in affected communities.^10,11^

We carried out Global Trachoma Mapping Project (GTMP)^12^-supported population-based trachoma prevalence surveys covering all suspected trachoma-endemic woredas without either (1) current control programs, or (2) population-based prevalence surveys in the 10 years preceding 2013. The aims were to estimate the prevalence of TF in children aged 1–9 years, and the prevalence of trichiasis in those aged 15 years and older, to guide future interventions in the region. We also collected water, sanitation and hygiene (WASH) data to evaluate risk factors for trachoma.

## Materials and methods

**Figure 1.**
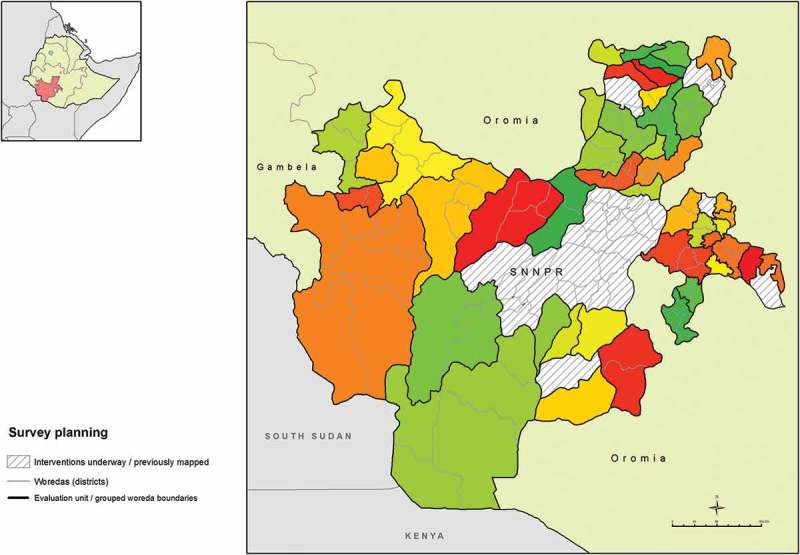
Evaluation units of grouped woredas (districts), Global Trachoma Mapping Project, Southern Nations, Nationalities, and Peoples’ Region, Ethiopia, 2013–2014.

Although collection of district-level data is generally recommended, in areas suspected to be highly and widely endemic for trachoma, WHO accepts that baseline surveys may be powered for larger populations, in order to expedite initiation of interventions where needed.^13^ Our first phase of surveys was designed to estimate prevalence in each of 37 evaluation units (EUs) consisting of 1–8 contiguous, grouped woredas, with those EUs therefore corresponding to sub-zones in the local administrative hierarchy. EUs were constructed in collaboration with local epidemiologists, respecting local administrative (zonal) boundaries. In one sub-zone-level EU, we did not find trachoma to be highly endemic (see below) and extra clusters were subsequently added in each of its four constituent woredas, in a second phase of fieldwork, in order to generate woreda-level estimates of trachoma prevalence in that sub-zone. Each EU comprised a total population of up to 250,000 inhabitants. The second phase split of one EU into four EUs ultimately generated data for a total of 40 EUs, covering 110 woredas over 16 zones (Figure 1). All sampling was carried out at EU level.

### Survey teams

Each team consisted of a trachoma grader, a data recorder and a driver. All graders and recorders had completed a standardized GTMP training course (version 1) and passed a formal examination.^14^ Training was conducted from 11 February 2013 to 15 February 2013. Trachoma graders were required to pass an inter-grader agreement test in the field against a GTMP-certified grader trainer. Recorders and graders were selected for training by the SNNPR Regional Health Office. Supervisors (normally ophthalmologists) who were also GTMP-certified trachoma graders or grader trainers, provided support to 3–4 teams throughout the mapping. Subsequent data processing and approval were undertaken as described elsewhere.^14^

### Survey design and sampling

The survey used a 2-stage cluster-randomized methodology outlined in full elsewhere,^14^ with specific refinements for use in SNNPR. The first stage cluster unit was the kebele. Kebeles were selected using census data obtained from the regional health bureau, and were randomly chosen with probability proportional to size. Before the survey was conducted, a representative of the regional health bureau went to each selected kebele, and one developmental unit (DU; a subdivision of a kebele with approximately 30 households) was selected randomly using the lottery method. DUs which had fewer than 26 households were excluded from the sampling frame. On the day of the survey, consent was initially obtained from the village leader, and the village health extension worker or the DU head was engaged to guide the teams and to introduce the team to each household.

### Sample size

As described elsewhere^14^ the sample size needed to estimate a 10% TF prevalence in children aged 1–9 years with an absolute precision of 3% at the 95% confidence level (and accounting for the non-independence of results in clustered sampling by using a design effect estimate from previous trachoma surveys of 2.65) was 1019 1–9-year-olds. This figure was inflated by 20% to accommodate non-response, giving an anticipated sample size of 1222 children aged 1–9 years. From the 2007 census data it was estimated that there would be on average 45 children per DU, therefore we needed to survey 26 such 30-household DU clusters in each EU.^9^

### Data collection

All household members at sampled households were eligible for inclusion. All consenting household members aged 1 year and older were examined for TF, TI and trichiasis using the WHO simplified trachoma grading system.^5,6^ Data recorders also collected global positioning system (GPS) coordinates of each sampled household, and WASH data by focused interview with the household head and direct observation of sanitation facilities. WASH variables collected were consistent with the WHO/UNICEF Joint Monitoring Programme outcomes.^14,15^ All data were collected using Android smartphones with the Task Force LINKS software (Task Force for Global Health, Decatur, GA, USA). Data were encrypted and uploaded to a secure cloud-based server when mobile network or Wi-Fi reception was available.

### Ethical considerations

Ethics clearance was obtained from SNNPR Health Bureau Ethical Review Committee, and the Ethics Review Committee of the Federal Ministry of Health of Ethiopia. Regional, zone and woreda officials were informed and provided with support letters for the study. In addition, ethics clearance was obtained from the Ethics Committee at the London School of Hygiene & Tropical Medicine (reference 6319). Verbal informed consent was obtained from the household head and electronically recorded in LINKS. All members of households found to have active trachoma were provided with a course of 1% tetracycline and instructions on its use. Those who needed further examination and treatment were referred to the nearest eye care center using a standardized referral form. Survey teams also provided general health education on limiting the spread of trachoma.

### Environmental risk factors

Cluster-level climatic data thought to be related to trachoma were obtained from WorldClim variables (worldclim.org),^16^ at a resolution of 2.5 arc-minutes (~5 km). Altitude was collected from global positioning system (GPS) data on smartphones at the time of survey. These measures were considered to possibly associate with likelihood of *C. trachomatis* transmission. Variables considered for models were altitude, annual mean temperature, mean annual precipitation, and maximum temperature in the hottest month.

### Data analysis

Briefly, TF estimates in the 1–9-year age range were adjusted for age in 1-year bands using the 2007 SNNPR census data.^9^ Trichiasis estimates were adjusted for both sex and age in 5-year bands. It was anticipated that by visiting 30 households per cluster, approximately the same number of participants would be examined per cluster. However, to account for natural variation in these numbers, clusters were equally weighted by adjusting at cluster-level and calculating the arithmetic mean of all such adjusted cluster estimates, with this mean used as the overall EU-level prevalence estimate.^14^ Confidence intervals (CIs) around these results were constructed by bootstrapping from these estimates and taking the 2.5th and 97.5th centiles of 10,000 bootstrap replicates.

Adjusted results and CIs were produced using R 3.0.2 (2013; The R Foundation for Statistical Computing, Vienna, Austria). Point values for climatic variables were extracted from rasters using ArcGIS 10.3 (Spatial Analyst; Environmental Systems Research Institute, Redlands, CA, USA). Risk factor analysis was carried out in Stata 10.2 (Stata Corp, College Station, TX, USA). Co-linearity was assessed using Mantel-Haenszel tests of association, but not used as an absolute exclusion criterion. A multi-level hierarchical model was used to account for clustering at kebele and household level. Univariable associations were assessed and variables were retained for the multivariable model if significant at the *p* < 0.05 level (Wald’s test). The full model was developed by a stepwise inclusion approach with variables retained if statistically significant at the *p* < 0.05 level (likelihood ratio test).

## Results

The first phase of 37 sub-zone-level surveys (covering 110 woredas) was carried out from 18 February to 10 June 2013. One EU, comprising four woredas (Gorchie, Hula, Malega, Wenesho) was found to have a TF prevalence between 5% and 10%, so in a second phase, these four woredas were further mapped as four EUs at woreda-level, using the same methodology previously applied at sub-zonal level. This meant adding clusters to each constituent woreda to reach the required sample size. We undertook this second phase from 3 April 2014 to 23 May 2014.

Overall, 14 rural zones of SNNPR were included in the surveys. A total of 40 EUs covering 113 woredas were ultimately surveyed. A total of 30,187 households were visited in 1047 kebeles, with 131,926 people enumerated, and 121,397 (92.0%) consenting to examination. Of those examined, 65,903 (54.3%) were female. There was a median of four inhabitants per household (interquartile range, IQR, 3–6), and a median of one child aged 1–9 years per household (IQR 0–2). The demographic characteristics of all individuals enumerated are outlined in Table 1.

**Table 1.** Demographic characteristics of individuals enumerated, Global Trachoma Mapping Project, Southern Nations, Nationalities, and Peoples’ Region, Ethiopia, 2013–2014.

Age group, years	Sex	Examined, *n* (%)	Absent, *n* (%)	Refused, *n* (%)	Other, *n* (%)	Total, *n*
1–9	Male	21,296 (96.2)	805 (3.6)	44 (0.2)	4 (0.0)	22,149
	Female	20,919 (97.5)	508 (2.4)	29 (0.1)	3 (0.0)	21,459
10–14	Male	8113 (88.1)	1074 (11.7)	21 (0.2)	0 (0.0)	9208
	Female	7871 (90.9)	761 (8.8)	23 (0.3)	2 (0.0)	8593
≥15	Male	26,085 (82.8)	5333 (16.9)	71 (0.2)	7 (0.0)	31,496
	Female	37,113 (95.3)	1767 (4.5)	73 (0.2)	4 (0.0)	38,957
Total		121,397	10,248	261	20	131,926

**Figure 2.**
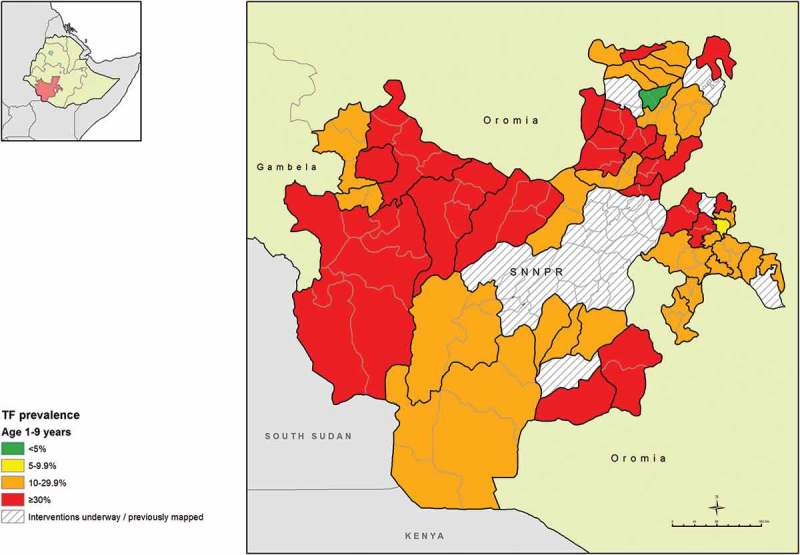
Prevalence of trachomatous inflammation – follicular (TF) in children aged 1–9 years, Global Trachoma Mapping Project, Southern Nations, Nationalities, and Peoples’ Region, Ethiopia, 2013–2014.

The age-adjusted EU-level prevalence of TF in children aged 1–9 years ranged from 2.3% (95% CI 0.6–4.8%) in Geta and Gumer woredas of Gurage Zone, to 48.5% (95% CI 41.5–55.9%) in Amaro and Burji in Segen Zone. There were only two EUs which had TF prevalences below 10%; Geta and Gumer, and Gorchie woreda in Sidama. All other EUs had TF prevalences ≥10% (Table 2, Figure 2).

**Table 2.** Trachomatous inflammation – follicular (TF) prevalence in children aged 1–9 years by evaluation unit, Global Trachoma Mapping Project, Southern Nations, Nationalities, and Peoples’ Region, Ethiopia, 2013–2014.

Zone	Evaluation unit	Examined, *n*	TF cases, *n*	Unadjusted TF, %	Adjusted^a^ TF, % (95% CI)
Hadiya	Misha, Anna Lemo, Gibe, Limo	1093	498	45.6	45.0 (38.1–51.8)
	Soro, Duna, Gombora	1092	397	36.4	36.0 (28.2–44.9)
	Shashego, Yem, Misrak Badawacho, Mirab Badawacho	1189	446	37.5	37.8 (31.6–44.8)
Sidama	Dale, Shebedino	837	273	32.6	30.6 (24.3–37.2)
	Hawassa Zuria, Boroche, Wenedo Genet	1036	424	40.9	39.5 (31.5–46.9)
	Arbegona, Bona Zuria, Bursa, Chirie	1238	251	20.3	17.4 (10.1–24.2)
	Dara, Aleta Wendo Town, Loka Abaya, Chiko	962	225	23.4	22.7 (16.7–29.3)
	Aroresa, Bensa	1120	324	28.9	27.7 (19.8–37.0)
	Gorchie	1029	68	6.6	6.3 (2.7–10.0)
	Hula	966	118	12.2	10.1 (5.4–15.0)
	Malega	1050	124	11.8	11.8 (7.2–15.3)
	Wenesho	900	150	16.7	13.6 (9.1–18.7)
Gedio	Kochere, Gedeb, Yergachefe	1299	251	19.3	18.3 (13.2–22.4)
	Bule, Wenago, Dilla Zuria	1105	209	18.9	15.0 (9.4–20.1)
South Omo	Basketo, South Ari, Semen Ari, Salamago, Debub Ari, North Ari	1072	304	28.4	26.6 (19.5–35.9)
	Hamer, Bena Tsemay, Dassenech, Malie, Nyngatom	1043	240	23	20.1 (13.3–27.8)
Sheka	Yeki, Masha Anderacha, Masha, Masha Town, Tepi Town	863	110	12.7	12.0 (6.2–18.3)
Kafa	Chena, Gesha, Gewata, Gimbo, Saylem	991	426	43.0	40.8 (32.9–48.9)
	Bita, Cheta, Decha, Menjiwo, Tello	1036	429	41.4	36.9 (29.1–44.5)
Bench Maji	Bero, Shay Bench, Surma, Guraferda, Maji, Meanit Goldiiya, Meint Shasha, Debub Bench	1115	398	35.7	32.0 (24.4–41.8)
	Semen Bench, Sheko, Mizan Aman Town	1212	358	29.5	28.5 (20.1–35.7)
Segen	Amaro, Burji	1301	658	50.6	48.5 (41.5–55.9)
East Gurage/Konta/Dawro	Konta, Isara, Tocha, Mareko	927	330	35.6	35.6 (27.2–44.9)
Dawro	Gena Bosa, Loma, Tercha Town	1030	303	29.4	28.1 (21.2–34.0)
Silti	Alicho Wuriro, Misirak Azerenert Berbere, Sankura, Mirab Azerenert Berbere, Wilbareg	984	315	32.0	26.4 (18.0–36.5)
	Dalocha, Lamfero, Selti	1191	368	30.9	27.0 (18.6–33.4)
Gamo Gofa	Kemba	1158	350	30.2	26.4 (19.1–35.5)
	Bonke	1098	193	17.6	15.8 (9.3–24.3)
	Arbaminch Town	1018	214	21.0	19.0 (13.5–25.5)
Special	Konso	1084	427	39.4	35.5 (28.2–41.9)
Kembata Tembaro/Halaba	Halaba Special woreda, Demboya, Kedida-Gamela	1166	524	44.9	41.0 (33.8–48.5)
	Angacha, Hadero Tunito, Kacha Bira, Doyugena, Tembaro	1005	323	32.1	29.1 (24.2–35.3)
Gurage	Cheha	794	256	32.2	29.1 (22.4–35.4)
	Ezha	798	120	15.0	13.9 (9.8–18.9)
	Mihur Aklil	784	234	29.8	27.6 (18.2–39.8)
	Kebena	959	286	29.8	30.8 (24.5–38.0)
	Kokir	958	157	16.4	15.4 (9.8–23.6)
	Abeshige	958	155	16.2	14.7 (9.6–20.2)
	Sodo	940	400	42.6	41.4 (31.2–49.9)
	Geta, Gumer	754	17	2.3	2.3 (0.6–4.8)

^a^Adjusted for age in 1-year age bands.

CI, confidence interval.

**Figure 3.**
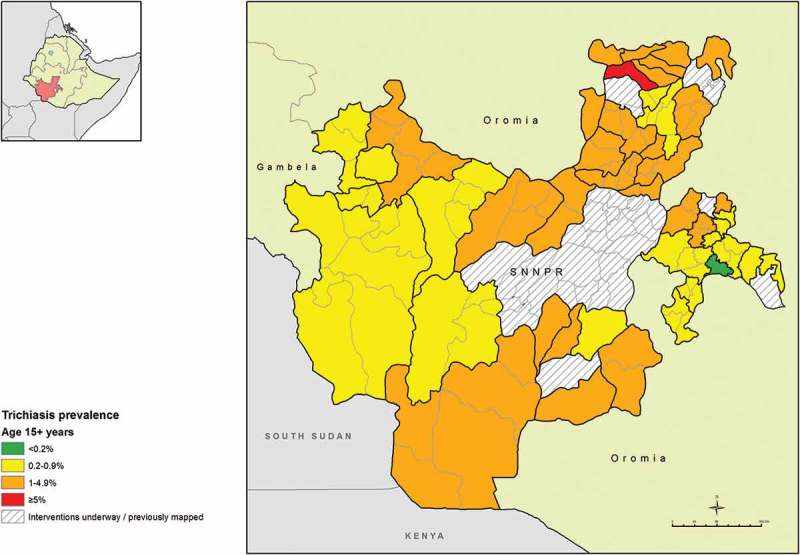
Prevalence of trichiasis in those aged ≥15 years, Global Trachoma Mapping Project, Southern Nations, Nationalities, and Peoples’ Region, Ethiopia, 2013–2014.

The age- and sex-adjusted prevalences of trichiasis in those aged 15 years and older ranged from 0.0% (95% CI 0.0–0.1%) in Hula woreda of Sidama Zone, to 6.1% (95% CI 4.4–7.8%) in Cheha in Gurage Zone. The trichiasis prevalence in those aged 15 years and older was 0.2% or less in three EUs covering six woredas in Sidama Zone; Arbergona, Bona Zuria, Bursa, Chirie, Gorchie and Hula (Table 3, Figure 3).

**Table 3.** Trichiasis prevalence in those aged ≥15 years by evaluation unit, Global Trachoma Mapping Project, Southern Nations, Nationalities, and Peoples’ Region, Ethiopia, 2013–2014.

		Examined age 15 years or greater			
Zone	Evaluation unit	Examined, *n*	Trichiasis cases, *n*	Unadjusted TT, %	Adjusted^a^ TT, % 95% CI
Hadiya	Misha, Anna Lemo, Gibe, Limo	1649	54	3.3	2.1 (1.3–2.7)
	Soro, Duna, Gombora	1674	74	4.4	2.6 (1.5–4.1)
	Shashego, Yem, Misrak Badawacho, Mirab Badawacho	1615	70	4.3	3.0 (2.0–4.2)
Sidama	Dale, Shebedino	1686	34	2.0	1.6 (0.9–2.5)
	Hawassa Zuria, Boroche, Wenedo Genet	1592	27	1.7	1.1 (0.5–1.9)
	Arbegona, Bona Zuria, Bursa, Chirie	1466	3	0.2	0.1 (0.0–0.1)
	Dara, Aleta Wendo Town, Loka Abaya, Chiko	1596	9	0.6	0.5 (0.1–1.0)
	Aroresa, Bensa	1558	9	0.6	0.4 (0.1–0.6)
	Gorchie	1506	2	0.1	0.1 (0.0–0.1)
	Hula	1478	2	0.1	0.0 (0.0–0.1)
	Malega	1370	7	0.5	0.3 (0.0–0.5)
	Wenesho	1528	8	0.5	0.3 (0.0–0.6)
Gedio	Kochere, Gedeb, Yergachefe	1614	17	1.1	0.8 (0.3–1.5)
	Bule, Wenago, Dilla Zuria	1456	22	1.5	0.7 (0.4–1.0)
South Omo	Basketo, South Ari, Semen Ari, Salamago, Debub Ari, North Ari	1205	13	1.1	0.6 (0.1–1.2)
	Hamer, Bena Tsemay, Dassenech, Malie, Nyngatom	1215	26	2.1	1.1 (0.5–1.5)
Sheka	Yeki, Masha Anderacha, Masha, Masha Town, Tepi Town	1987	21	1.1	0.7 (0.3–1.1)
Kafa	Chena, Gesha, Gewata, Gimbo, Saylem	1727	25	1.4	1.1 (0.5–1.9)
	Bita, Cheta, Decha, Menjiwo, Tello	1592	22	1.4	0.9 (0.5–1.4)
Bench Maji	Bero, Shay Bench, Surma, Guraferda,Maji, Meanit Goldiiya, Meint Shasha, Debub Bench	1306	11	0.8	0.6 (0.2–1.1)
	Semen Bench, Sheko, Mizan Aman Town	1470	15	1.0	0.8 (0.3–1.3)
Segen	Amaro, Burji	1500	59	3.9	2.6 (1.6–3.4)
East Gurage/Konta/Dawro	Konta, Isara, Tocha, Mareko	1462	58	4.0	2.3 (1.4–3.1)
Dawro	Gena Bosa, Loma, Tercha Town	1620	34	2.1	1.2 (0.8–1.7)
Silti	Alicho Wuriro, Misirak Azerenert Berbere, Sankura, Mirab Azerenert Berbere, Wilbareg	1434	27	1.9	0.8 (0.4–1.4)
	Dalocha, Lamfero, Selti	1468	52	3.5	2.0 (1.3–2.7)
Gamo Gofa	Kemba	1456	28	1.9	1.1 (0.5–1.8)
	Bonke	1481	27	1.8	1.2 (0.7–1.9)
	Arbaminch Town	1416	31	2.2	0.8 (0.5–1.2)
Special	Konso	1394	33	2.4	1.0 (0.7–1.4)
Kembata Tembaro/Halaba	Halaba Special woreda, Demboya, Kedida-Gamela	1559	53	3.4	2.0 (1.3–2.7)
	Angacha, Hadero Tunito, Kacha Bira, Doyugena, Tembaro	1640	77	4.7	3.1 (2.3–3.8)
Gurage	Cheha	1606	184	11.5	6.1 (4.4–7.8)
	Ezha	1895	97	5.1	2.2 (1.4–3.3)
	Mihur Aklil	1488	98	6.6	4.3 (2.8–6.3)
	Kebena	1546	125	8.1	4.5 (3.1–6.2)
	Kokir	1425	35	2.5	1.6 (1.0–2.5)
	Abeshige	1730	80	4.6	3.3 (2.2–4.5)
	Sodo	1473	49	3.3	1.4 (0.7–2.0)
	Geta, Gumer	1830	17	0.9	0.3 (0.1–0.6)

Adjusted for sex and age in 5-year age bands.

CI, confidence interval.

### Multilevel clustering of TF and TI

Null models for both TF and TI adjusted for age and sex showed statistically significant clustering at EU, kebele (cluster) and household levels. For TF, the estimated standard deviation for between-EU clustering was 2.78 (standard error, SE, 1.13, *p* < 0.0001), for between-kebele clustering was 4.08 (SE 1.04, *p* < 0.0001), and for between-household clustering was 3.0 (SE 1.03, *p* < 0.0001). For TI, the estimated standard deviation for between-EU clustering was 2.0 (SE 1.1, *p* = 0.001), for between-kebele clustering was 3.17 (SE 1.06, *p* < 0.0001), and for between-household clustering was 3.16 (SE 1.06, *p* < 0.0001). For both TF and TI, clustering was strongest at kebele level, and all subsequent analyses were conducted as 2-level hierarchical regression models with adjustment for the kebele inhabited by examined individuals.

### Risk factors and climatic variables

Climatic data were extracted from the raster data for all 1047 clusters. The median annual mean temperature over all clusters was 18.0°C (IQR 16.0–19.4°C), the median annual maximum temperature was 27.2°C (IQR 24.7–28.8°C), and the median annual mean precipitation was 1240mm (IQR 1124–1453mm). The median cluster-level altitude was 1984m (IQR 1769–2427m).

The full results of univariable analysis are shown in Table 4. In the full multivariable model (Table 5), factors independently associated with TF and TI in children aged 1–9 years were being a younger child, living at an altitude <2500m, and living in an area where the annual mean temperature was above 15°C. Additionally, the use of open defecation by adult household members was associated with TF, but not TI, in children aged 1–9 years.

**Table 4.** Multilevel univariable analysis of factors related to trachomatous inflammation – follicular (TF) and trachomatous inflammation – intense (TI) in children aged 1–9 years, Global Trachoma Mapping Project, Southern Nations, Nationalities, and Peoples’ Region, Ethiopia, 2013–2014.

		TF		TI	
Variable	Examined, *n*	%	Odds ratio^a^ (95% CI)	%	Odds ratio^a^ (95% CI)
*Individual*					
Age, years					
1–4	17,649	36.4	**2.66 (2.53–2.81)**	5.3	**2.40 (2.16–2.68)**
5–9	25,960	20.5	1 (reference)	2.4	1 (reference)
Sex					
Male	22,149	26.9	1 (reference)	3.5	1 (reference)
Female	21,459	27.0	1.01 (0.97–1.06)	3.7	0.89 (0.77–1.02)
*Household*					
Children aged 1–9 years in household, *n*					
1–3	34,061	27.2	1 (reference)	3.7	1 (reference)
≥4	9547	26.0	1.02 (0.96–1.09)	3.3	0.89 (0.78–1.02)
Inhabitants in household, *n*					
1–7	36,477	27.8	1 (reference)	3.6	1 (reference)
≥8	7131	22.6	0.91 (0.85–1.02)	3.2	0.97 (0.82–1.14)
Time to main source of drinking water, minutes					
<30	20,378	26.7	1 (reference)	3.8	1 (reference)
≥30	23,230	27.2	0.97 (0.90–1.05)	3.4	0.94 (0.81–1.09)
Time to main source of water used for face-washing, minutes					
All washing at source	112	28.6	1.02 (0.43–2.39)	6.3	2.81 (0.69–11.27)
<30	21,996	26.4	1 (reference)	3.8	1 (reference)
≥30	21,500	27.4	1.00 (0.93–1.08)	3.3	0.90 (0.77–1.04)
Surface water^b^ used as drinking source					
Yes	5296	30.5	1.19 (1.03–1.38)	4.6	1.21 (0.95–1.55)
No	38,312	26.4	1 (reference)	3.4	1 (reference)
Surface water^b^ used for face-washing					
Yes	8018	28.4	**1.11 (0.99–1.24)**	4.5	**1.29 (1.06–1.58)**
No	35,590	26.6	1 (reference)	3.4	1 (reference)
Latrine type^c^					
Improved latrine	2367	23.8	1 (reference)	3.0	1 (reference)
Unimproved latrine	33,937	26.3	**1.07 (0.93–1.22)**	3.5	**1.10 (0.83–1.46)**
Open defecation (no facilities, bush, or field)	7304	31.1	**1.18 (1.02–1.37)**	4.1	**1.28 (0.95–1.76)**
*Kebele (cluster)*					
Altitude^d^, m above sea level					
<2500	34,696	31.5	1 (reference)	4.3	1 (reference)
≥2500	8883	8.9	**0.21 (0.17–0.25)**	0.8	**0.18 (0.13–0.24)**
Mean annual precipitation^e^, mm					
<1000	6403	35.4	**2.01 (1.54–2.63)**	4.7	**1.66 (1.23–2.24)**
1000–1499	27,968	24.5	1 (reference)	3.2	1 (reference)
≥1500	9237	28.5	**1.41 (1.13–1.77)**	4.0	**1.44 (1.11–1.85)**
Mean annual temperature^e^, ◦C					
<15	7724	7.0	**0.10 (0.08–0.13)**	0.8	**0.16 (0.11–0.23)**
≥15	35,884	31.2	1 (reference)	4.2	1 (reference)
Maximum temperature of warmest month^e^, °C					
<25	11,150	12.3	**0.18 (0.15-0.22)**	1.2	**0.23 (0.18-0.31)**
≥25	32,458	32.0	1 (reference)	4.4	1 (reference)

^a^Multilevel univariable random effects regression accounting for clustering at kebele-level, with 95% CI; *p*-value from Wald’s test; variables significant at the *p* < 0.10 level are shown in bold.

^b^River, dam, lake, canal.

^c^WHO/UNICEF Joint Monitoring Programme improved sanitation definitions; open defecation displayed separately.

^d^Estimate from GPS coordinates of household measured on smartphones at the time of survey.

^e^Estimate from GPS coordinates of household, extracted at kebele(cluster)-level from Bioclim rasters (Worldclim.org).

CI, confidence interval; WHO, World Health Organization; GPS, global positioning system.

**Table 5.** Multivariable multi-level risk factor model for the outcomes trachomatous inflammation – follicular (TF) and trachomatous inflammation – intense (TI) in children aged 1–9 years, Global Trachoma Mapping Project, Southern Nations, Nationalities, and Peoples’ Region, Ethiopia, 2013–2014.

Variable	TF model		TI model	
	Odds ratio^a^ (95% CI)	*P*-value^b^	Odds ratio^a^ (95% CI)	*P*-value^b^
Age, years		<0.0001		<0.0001
1–4	2.65 (2.52–2.79)		2.38 (2.13–2.65)	
5–9	1 (reference)		1 (reference)	
Mean annual temperature^c^, °C		<0.0001		0.001
<15	0.16 (0.11–0.23)		0.42 (0.25–0.69)	
≥15	1 (reference)		1 (reference)	
Altitude^d^, metres above sea level		<0.0001		<0.0001
<2500	1 (reference)		1 (reference)	
≥2500	0.50 (0.40–0.70)		0.31 (0.20–0.49)	
Latrine type^e^		0.0311		0.1163^f^
Improved latrine	1 (reference)		1 (reference)	
Unimproved latrine	1.10 (0.96–1.26)		1.17 (0.88–1.56)	
Open defecation (no facilities, bush, or field)	1.19 (1.02–1.38)		1.33 (0.97–1.82)	

^a^Multilevel multivariable random effects regression accounting for clustering at kebele-level. Sex was included in the model *a priori*, but was not statistically significant for either TF or TI (*p* < 0.05 level, likelihood ratio test).

^b^Likelihood ratio test.

^c^Estimate from GPS coordinates of household, extracted at kebele-level from Bioclim rasters (Worldclim.org).

^d^Estimate from GPS coordinates of household measured on smartphones at the time of survey.

^e^WHO/UNICEF Joint Monitoring Programme improved sanitation definitions; open defecation displayed separately.

^f^Not included in final TI model, but presented for illustrative purposes.

CI, confidence interval; WHO, World Health Organization; GPS, global positioning system.

## Discussion

Trachoma is clearly a major public health problem in SNNPR. A total of 38 of 40 EUs surveyed had TF prevalences above the 10% threshold at which WHO recommends population-level distribution of antibiotics for 3 years before re-survey. One of 37 sub-zone-sized EUs had a TF prevalence between 5.0% and 9.9% in the first phase, and was re-mapped at woreda level in the second phase; three of four constituent woredas had TF prevalences above 10%, while the fourth had a TF prevalence between 5.0% and 9.9%. The final EU (composed of two woredas) had a TF prevalence of 2.3%, below the elimination threshold for TF. This means that in SNNPR, 108 woredas require mass drug administration (MDA) with azithromycin for at least 3 years, plus implementation of the F and E components of the SAFE strategy, before impact surveys. Overall, 15 of the 38 EUs (48 woredas) had TF prevalences above 30% and will require MDA for at least 5 years before impact surveys are needed.

The reason for the density of hyper-endemic woredas in SNNPR is unclear, and the risk factor analysis did not help much to generate hypotheses. Younger children have been associated with higher odds of trachoma in many previous studies.^17–19^ In addition, open defecation is thought to increase infection transmission by providing an ecological niche for eye-seeking flies known to carry *C. trachomatis*.^20,21^ Trachoma has been associated with altitude before,^21^ but the finding in these surveys of a lower odds of trachoma at high (≥2500m) altitudes is difficult to explain. It has previously been suggested that at higher altitudes, lower temperatures decrease fly activity,^22^ but why this would cause an effect independent of temperature at low altitude is unclear. It is possible that lower altitudes in SNNPR provide a climate where people are more likely to settle, and so human population density may be an important association in these areas.

The prevalence of trichiasis throughout SNNPR was high. In 37 of 40 EUs (104 woredas) the prevalence was higher than the elimination prevalence target (set by WHO) of <0.2% in the population 15 years of age and older.^23^ It is likely that without robust interventions being rapidly put in place, many people will go blind in SNNPR in the coming years as they wait for surgery. This warrants the development and maintenance of a strong program that will address the backlog of patients with trichiasis. To illustrate the magnitude of the undertaking required, 184 cases of trichiasis were identified in the EU covering Cheha woreda alone, giving an estimated trichiasis prevalence of 6.1% of the adult population there. Cheha has been known as a trachoma hotspot for many years, and eye care NGOs have been targeting trichiasis surgery outreach programs there since as early as 2006, yet even now, the prevalence is still among the highest trichiasis prevalence estimates ever reported. The reason that Cheha should have such a large trichiasis burden is unclear, but research may be helpful to guide the regional program as it seeks to recruit, train and deploy trichiasis surgeons to address it, and to understand the barriers that people in these areas have to seeking healthcare.

Our use of EUs generally comprising multiple woredas allowed us to more rapidly complete baseline mapping of SNNPR than if each woreda had been approached as an individual EU. However, as interventions decrease TF prevalence, it is likely that impact surveys will need to be carried out at higher resolution, in other words, at woreda-level. This will require a significant increase in funding to support the trachoma elimination effort in SNNPR.

Active and potentially blinding trachoma are highly prevalent throughout SNNPR, and in 108 woredas the disease presents a significant public health problem. Political support, coordination and funding for trachoma elimination are all urgently required.

## Appendix

The Global Trachoma Mapping Project Investigators are: Agatha Aboe (1,11), Liknaw Adamu (4), Wondu Alemayehu (4,5), Menbere Alemu (4), Neal D. E. Alexander (9), Berhanu Bero (4), Simon J. Brooker (1,6), Simon Bush (7,8), Brian K. Chu (2,9), Paul Courtright (1,3,4,7,11), Michael Dejene (3), Paul M. Emerson (1,6,7), Rebecca M. Flueckiger (2), Allen Foster (1,7), Solomon Gadisa (4), Katherine Gass (6,9), Teshome Gebre (4), Zelalem Habtamu (4), Danny Haddad (1,6,7,8), Erik Harvey (1,6,10), Dominic Haslam (8), Khumbo Kalua (5), Amir B. Kello (4,5), Jonathan D. King (6,10,11), Richard Le Mesurier (4,7), Susan Lewallen (4,11), Thomas M. Lietman (10), Chad MacArthur (6,11), Colin Macleod (3,9), Silvio P. Mariotti (7,11), Anna Massey (8), Els Mathieu (6,11), Siobhain McCullagh (8), Addis Mekasha (4), Tom Millar (4,8), Caleb Mpyet (3,5), Beatriz Muñoz (6,9), Jeremiah Ngondi (1,3,6,11), Stephanie Ogden (6), Alex Pavluck (2,4,10), Joseph Pearce (10), Serge Resnikoff (1), Virginia Sarah (4), Boubacar Sarr (5), Alemayehu Sisay (4), Jennifer L. Smith (11), Anthony W. Solomon (1,2,3,4,5,6,7,8,9,10,11), Jo Thomson (4); Sheila K. West (1,10,11), Rebecca Willis (2,9).

Key: (1) Advisory Committee, (2) Information Technology, Geographical Information Systems, and Data Processing, (3) Epidemiological Support, (4) Ethiopia Pilot Team, (5) Master Grader Trainers, (6) Methodologies Working Group, (7) Prioritisation Working Group, (8) Proposal Development, Finances and Logistics, (9) Statistics and Data Analysis, (10) Tools Working Group, (11) Training Working Group.
